# Role of natural gas and nuclear energy consumption in fostering environmental sustainability in India

**DOI:** 10.1038/s41598-023-38189-4

**Published:** 2023-07-07

**Authors:** Tomiwa Sunday Adebayo, Ilhan Ozturk, Mehmet Ağa, Solomon Eghosa Uhunamure, Dervis Kirikkaleli, Karabo Shale

**Affiliations:** 1 Department of Business Administration, Faculty of Economics and Administrative Science, Cyprus International University, Northern Cyprus, Mersin-10, 99040 Nicosia, Turkey; 2 Department of Economic and Data Sciences, New Uzbekistan University, 54 Mustaqillik Ave, 100007 Tashkent, Uzbekistan; 3 College of Business Administration, University of Sharjah, Sharjah, UAE; 4 Faculty of Economics, Administrative and Social Sciences, Nisantasi University, Istanbul, Turkey; 5 Department of Medical Research, China Medical University Hospital, China Medical University, Taichung, Taiwan; 6 Department of Finance and Banking, European University of Lefke, North Cyprus, Mersin 10 Turkey; 7 Faculty of Applied Sciences, Cape Peninsula of Technology, P. O. Box 652, Cape Town, 8000 South Africa; 8 Department of Banking and Finance, Faculty of Economic and Administrative Sciences, European University of Lefke, Via Mersin, Lefke/Northern Cyprus, Turkey

**Keywords:** Environmental biotechnology, Climate sciences, Environmental sciences, Environmental social sciences, Nuclear energy, Renewable energy

## Abstract

This paper investigates the role of nuclear energy in promoting ecological sustainability in India, focusing on three ecological indicators: ecological footprint (EF), CO2 emissions (CO_2_), and load capacity factor (LF). In addition to nuclear energy, the study considers the influence of gas consumption and other drivers of ecological sustainability using data spanning from 1970 to 2018. The analysis also takes into account the impact of the 2008 global financial crisis on the model, employing the autoregressive distributed lag (ARDL) and frequency domain causality approaches to assess the relationships. Unlike previous studies, this research evaluates both the Environmental Kuznets Curve (EKC) and load capacity curve (LCC) hypotheses. The ARDL results support the validity of both the EKC and LCC hypotheses in the Indian context. Furthermore, the findings reveal that nuclear energy and human capital contribute positively to ecological quality, while gas consumption and economic growth have a negative impact on ecological sustainability. The study also highlights the increasing effect of the 2008 global financial crisis on ecological sustainability. Additionally, the causality analysis demonstrates that nuclear energy, human capital, gas consumption, and economic growth can serve as predictors of long-term ecological sustainability in India. Based on these findings, the research presents policy recommendations that can guide efforts towards achieving SDGs 7 and 13.

## Introduction

Human consumption of services and goods places strain on the environment, which is the source of modern threats such as ecological distortions, climate change and environmental destruction^1–3^. As a result, environmentalists and economists have made global awareness and commitment toward environmental protection and sustainable development their primary focus. The majority of contemporary research employs CO_2_ as a proxy for environmental deterioration in ecological assessment^4–8^. Nevertheless, some recent investigations favour a new ecological measure, such as the load capacity factor (LF). This measure takes into account nations’ ecological footprint (EF) and biocapacity. As a result, the literature on ecological sustainability and its drivers has grown in size.

**Figure 1:**
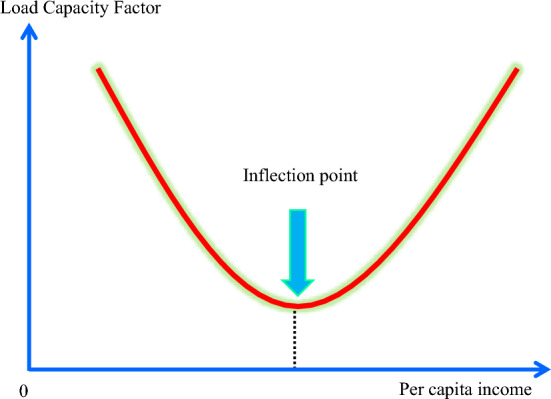
LCC curve. Source: Adapted from the study of^11^.

Substantial studies have concentrated on evaluating the EKC presented by^9^ when engaging with ecological sustainability. Ecological footprint (EF) and CO_2_ are frequently utilized as dependent indicators in these investigations, but the supply side of ecological issues is ignored. EF elements in EF accounting represent the demand of humans for natural resources, while the quantity of prevailing natural resources that can fulfil the demand is represented by biocapacity. Thus, the LF introduced by^10^ permits ecological evaluation from both demand and supply dimensions. The study on LF is expanding but not yet completely developed. The EKC represents a U-shaped inverted interconnectedness between ecological deterioration and income. This research not only validates the EKC concept but also provides an innovative curve termed "Load Capacity Curve (LCC)". Prior research on the factors of LF has mostly relied on linear models. On contrary, this research suggests that a U-shaped non-linear interrelationship between income and LF may exist, and the “U-shaped curve” is referred to as LCC (see Fig. 1).

Figure 1 demonstrates that as income rises, environmental quality (LF) first falls, however, after reaching a threshold of income, LF rises due to the development of green technologies and ecological awareness. This curve is the EKC’s exact inverse which is known as load capacity curve hypothesis^11^. According to the LCC hypothesis, increased income can impact both EF and biocapacity (BIO). The LCC suggests that in the initial phases of economic expansion, ecological integrity is substantially harmed by the use of fossil fuel-based energy due to neglect of the ecosystem condition. Conversely, when income exceeds a specific threshold, people consume more eco-friendly items and employ clean sources of energy, so ecological sustainability is enhanced by decreasing EF and boosting BIO.

The emergence of COVID-19 in late 2019 contributed significantly to the reduction of environmental pollutants. However, the restriction imposed by various nations such as India has led to massive economic loss; nonetheless, it has come as a relief to the environment. Furthermore, the recent Ukraine-Russia war has triggered an energy crisis and as a result several nations mostly the European nations are shifting their agenda to nuclear energy and a source of energy which is eco-friendly when properly managed.

Energy is the main input in the manufacturing progression and is used as both labour and capital^12,13^. Though energy is necessary for sustainable economic growth, as demonstrated by Refs.^14,15^, it also contributes significantly to ecological damage. Modernization of energy processes may aid in pollution reduction^16–18^. Furthermore, at the moment, electricity production is heavily reliant on fossil fuels. More nuclear energy may be used to generate electricity, which can help to mitigate environmental issues^17,19,20^.

Nuclear energy is intended to be an alternative resource to deal with rising oil costs and reduce reliance on other countries for energy needs. Nuclear power plants are capital-intensive, and nuclear energy charges are more sensitive to changes in fuel prices than gasoline or coal consumption. Furthermore, nuclear energy is an important resource in environmental measures and energy development that can help to alleviate the long-term climate change effects^21,22^. Nuclear energy has surfaced as an alternative energy source to meet energy needs in several nations globally where energy demand is increasing, gas and oil stocks are predicted to deplete in the years ahead, and power delivery protection is the top consideration, and mitigating GHGs emissions and air pollution is part of environmental and economic policies^11,22,23^.

In reaction to these challenges, nuclear energy plays an important role in long-term environmental and development goals. With expanding electricity consumption, it meets global energy demands^24^. On the opposite, the expansion of nuclear energy faces some problems, such as a system to eradicate radioactive wastes, safe operations, the risk associated with the construction of nuclear apparatus, and negative popular sentiment and civil society criticism of nuclear energy^17,25^. Nuclear energy is capable of generating electricity and is generally useful in alleviating environmental issues^20^. It is a suitable and well-known resource in support of minimizing carbon output. On the other hand, due to the adverse ecological effects of atomic disasters and radioactive waste, nuclear energy has not been employed to support CO_2_ reduction efforts^26,27^. It damages the ecosystem and more radioactive waste harms the ecosystem and people. The production of radioactive garbage, such as aged reactor fuel, uranium grind tailings, and other radioactive garbage, is a serious ecological issue associated with nuclear power. Such resources may emit hazardous radioactive waves that can be lethal to humans for thousands of years.

Human actions, as per^28^, have an adverse influence on the ecosystem, degrade water quality and reduce production size. As a result, the advancement of human capital (HC) may have an impact on these operations, which are connected to issues of environmental degradation and energy security. HC can influence ecological deterioration and energy security. Furthermore, it can impair humans' capacity to manage their workplaces effectively^29^. Human capital has proven to be an important factor in reducing tailpipe emissions by enhancing the efficiency of energy^30^. HC also increases individual efficiency through modern manufacturing procedures. It boosts the willingness of economies to create and invest in contaminant-free technologies, notably in the industry, household, and transport sectors^31–33^. Examining the impact of human capital on ecological quality may help countries achieve their long-term financial development goals^34^. The optimal use of natural resources and usage of energy is connected to human capital training and skills^35^. Works have demonstrated the efficacy of HC in promoting ecological sustainability by reducing dependency on non-renewable sources of energy (e.g., gas, oil, and coal). Following this viewpoint, this analysis considered HC as a predictor of environmental sustainability.

**Figure 2:**
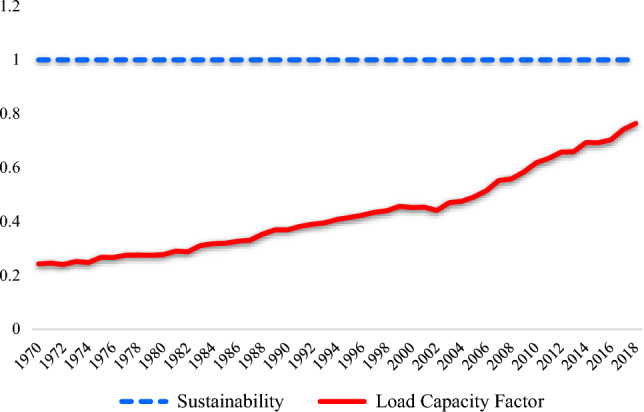
Evolution of Ecological Situation in India. Source: GFN (2022).

Over the years, substantial works have been conveyed concerning the nexus between NC and various ecological quality/degradation proxies in the top nuclear nations such as China, the USA, France, South Korea, Russia, and Canada which account for roughly 74.2% of the global nuclear energy use globally. India is placed 14^th^ in the global ranking with nuclear power consumption with nuclear electricity supplied approximately 39,758 GW-HR in 2021. Prior studies^17,17,23,25,26^ have extensively inspected the role of nuclear energy in the top nuclear consuming and generating countries such as France, USA, China, and South Korea. Nonetheless, scant scholars have taken a keen interest in investigating the role of nuclear energy towards carbon neutrality in the case of India. As a result, research on the Indian situation can add to the body of knowledge. Figure 2 depicts the evolution of ecological indicators in India.

**Figure 3:**
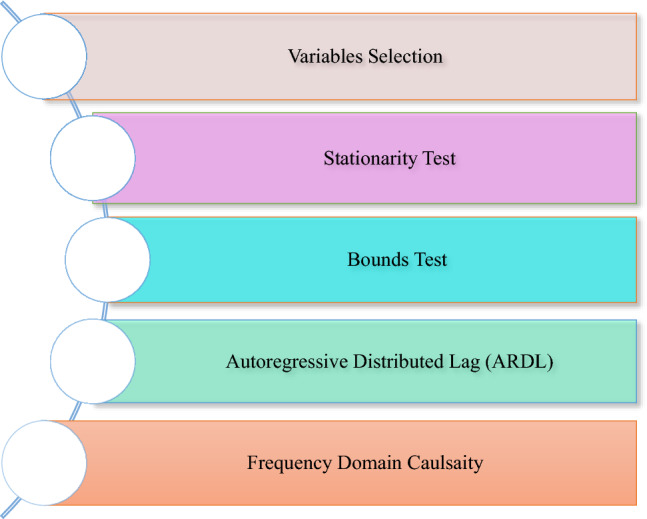
Flow of the study.

As can be observed in Fig. 3, an increase in EF in India is accompanied by a decrease in biocapacity. Furthermore, the load capacity factor value is still under 1, which indicates that the ecological situation in India is unsustainable. Although the LF of India per person has gradually increased, specifically from 0.24 in 1970 to 0.76 in 2018, the country is still experiencing several ecological issues since it has not reached the threshold sustainability value of 1. Although various studies have analyzed the LF viewpoint in the existing literature, no research has thus far evaluated the Indian situation by examining the nexus between NC and LF.

In summary, although research on the LF is expanding, available studies remain limited. Some investigations have discussed the Indian case, but none have evaluated it from the perspective of LF and NC in single research. As a result, no previous studies have evaluated the Indian example in the LF scenario extensively and utilized the most recent data. Given the literature deficiency, India's position as one of the main nuclear consumers, and the possible contribution of NC to ecological quality (EQ), this paper evaluates the effects of NC on EQ proxies i.e., LF, CO_2_, and EF, while also considering the role of gas consumption, human capital, and the 2008 financial crisis. The research objectives are structured as follows: (a) the legitimacy of both LCC and EKC hypotheses are tested; and (b) the role of NC on LF, CO_2_ and EF in India is evaluated taking into account gas consumption, human capital, and the 2008 financial crisis.

This paper renders substantial addition to prior investigations. Firstly, this paper concentrates on India as a nuclear energy-consuming nation. Though studies have been conveyed in India^36–38^; however, these studies neglect LF. Secondly, this paper distinct itself from the prior study by incorporating three EQ proxies (i.e., EF, LF and CO_2_) to evaluate the LCC and EKC hypotheses. Given that the research on the LF is expanding but not yet mature, this research can be regarded as pioneer research because it draws on the India case. Thirdly, this paper considers nuclear energy, gas consumption, human capital, and the 2008 financial crisis as explanatory variables. Lastly, unlike prior studies, that use time—domain causality, the current research utilised frequency—domain causality to identify causality between LF and the independent variables at dissimilar frequency.

The research is divided into 5 sections. “Literature review” presents a synopsis of related studies. “Data, model and methodology” presents the model, and methods. “Findings and discussion” presents the results which are accompanied by the discussions. “Conclusion and policy recommendations” concludes the investigation.

## Literature review

This section presents a synopsis of past studies. Over the years, studies on the role of NC on EQ have been conducted. However, these studies^20,22,23,26,39^ have produced mixed results. For example^26^, investigated the interrelationship between NC and EQ using CVAR in nuclear-generating nations. The research found that NC causes EQ in all nations. Similarly^20^, inspected the association between EQ and NC in the United States from 1960 to 2007. The research found a unidirectional causality going from NC to EQ without feedback utilizing a revamped version of the causality. Likewise^40^, investigated the NC-EQ connection in 12 developed markets from 1980 to 2015. The results reveal that the estimation of NC is significantly negative, showing that NC can help the environment by protecting land, forest resources and water and improving EQ. Moreover^22^, analyse the NC-EQ linkage in 15 OECD nations from 1990 to 2018. The FMOLS findings indicate that investing in NC enhances EQ.

Human capital (HC) influences energy security and environmental problems, as well as people's capacity to complete their workstations successfully^41^. HC is critical in increasing EQ through increasing energy efficiency^42^. Furthermore, to care for the environment,^35^ inspected the HC and EQ nexus in BRICS using data from 1991 to 2019 with results suggesting that HC boosted EQ^43^ analysed the HC-EQ interconnection for the NICs from 1979 to 2017. The results from the DOLS and PMG methods provide similar results exhibiting that HC boosts EQ in the selected nations. Moreover, a pathway towards a sustainable ecosystem in developing nations was explored by^34^ using CSARDL from 1995 to 2018. The study explores the role of HC on EQ and the study result discloses that EQ can be attained by increasing investment in HC. The criticality of HC in EQ is examined by^44^ in the Caribbean and Latin American countries using CUP-BC and CUP-FM long-run estimators between 1995 and 2017 with the results suggesting that HC reduces EQ.

Gas consumption is one of the leading causes of economic growth and an upsurge in ecological deterioration degradation. Moreover,^45^ ask the question “does gas consumption increase EQ” using Asia–Pacific countries from 1970 to 2016. The result uncovers that gas consumption impact EQ positively; the significant and positive effect of GAS on EQ is also independent of economic expansion but, conversely, may be impacted by the natural gas proportion in the primary energy mix. Using the nonlinear ARDL, gas consumption and EQ nexus in the USA were inspected by^46^ from 1997 to 2017. The cointegration test findings demonstrate that gas consumption has a long-run interrelationship with EQ in the eight states studied. Furthermore, the findings show that the asymmetric influence of natural gas usage on EQ varies by state.

### Contribution of the study

Despite the extensive research on the impact of gas consumption and human capital on ecological sustainability, there is only a limited body of literature that has comprehensively explored nuclear energy consumption (NC) within the framework of the load capacity factor (LF) paradigm. Furthermore, existing studies have primarily focused on major nuclear energy-consuming and producing countries such as South Korea, France, China, the United States, and Russia. Hence, there is a notable research gap, as no investigation in the literature has specifically examined the effects of NC on ecological quality (EQ) in India from the perspective of LF. Hence, this research aims to contribute to the existing literature by adopting a comprehensive approach to analyze the relationship between NC and EQ in India. By filling this research gap, the study not only provides insights for nuclear energy-producing nations, but also offers policy implications for countries considering nuclear energy as a potential solution to address energy crises and ecological degradation. Through this holistic analysis, the research intends to shed light on the unique dynamics and implications of NC in the Indian context, emphasizing the importance of considering LF as a key factor in understanding the relationship between nuclear energy consumption and ecological sustainability. The findings of this study will contribute to the knowledge base and inform policymakers and stakeholders in both nuclear energy-producing nations and those exploring the potential of nuclear energy in tackling energy and environmental challenges.

## Data, model and methodology

### Data

The paper employs yearly data spanning between 1970 and 2018 for India to evaluate the impact of gas consumption (GAS), nuclear energy (NC), economic growth (EG) and human capital (HC) on three distinct environmental quality proxies (load capacity factor (LF), ecological footprint (EF) and CO_2_ emissions (CO_2_). The study's timeline was limited to the period from 1970 onwards due to the unavailability of natural gas data before that year. Similarly, the study concluded in 2018 due to the lack of comprehensive ecological footprint and load capacity data beyond that year. These data limitations restricted the analysis to the available data timeframe. The dependent variables are LF, EF and CO_2_ while the regressors are NC, EG, HC and GAS. Since this LF contains EF in the denominator and biocapacity in the numerator, it enables ecological evaluation on both the demand and supply sides. A higher LF demonstrates a more conducive ecosystem. The indicators of examination are logged to guarantee they conform to the normal distribution in line with the study of^47^. Table 1 presents concrete information on the source, measurement and variables employed.

**Table 1:** Variables sign, measurement and source.

Sign	Variables	Measurement	Source
GAS	Gas consumption	Exajoules	BP database
NC	Nuclear energy	Exajoules	BP database
EG	Economic growth	GDP per capita	WB database
HC	Human capital	Index	Pen database
CO_2_	CO2 emissions	Per capita	Ourworldindata.org database
EF	Ecological footprint	Global hectares per capita	GFN database
LF	Load capacity factor		GFN database

### Model and theoretical framework

The LCC and EKC hypotheses are examined using three distinct frameworks in the research. To avert the heteroscedasticity issue and to evaluate elasticities, all indicators are incorporated into Eqs. (1), (2), and (3) with logarithmic conversions.

1 $${InCO}_{2t}={\beta }_{0}+{\beta }_{1}{InEG}_{t}+{\beta }_{2}{InEGSQ}_{t}+{\beta }_{3}{InNC}_{t}+{\beta }_{4}{HC}_{t}+{\beta }_{5}{GAS}_{t}+{\varepsilon }_{t}$$

2 $${InEF}_{t}={\gamma }_{0}+{\gamma }_{1}{InEG}_{t}+{\gamma }_{2}{InEGSQ}_{t}+{\gamma }_{3}{InNC}_{t}+{\gamma }_{4}{HC}_{t}+{\gamma }_{5}{GAS}_{t}+{\rho }_{t}$$

3 $${InLF}_{t}={\vartheta }_{0}+{\vartheta }_{1}{InEG}_{t}+{\vartheta }_{2}{InEGSQ}_{t}+{\vartheta }_{3}{InNC}_{t}+{\vartheta }_{4}{HC}_{t}+{\vartheta }_{5}{GAS}_{t}+{\sigma }_{t}$$

In equations above, the constant terms are shown by ${\beta }_{0}$, ${\gamma }_{0}$ and ${\vartheta }_{0}$. Furthermore ${\beta }_{\mathrm{1,2},\mathrm{3,4} and 5}$, ${\gamma }_{\mathrm{1,2},\mathrm{3,4} and5}$ and ${\vartheta }_{\mathrm{1,2},\mathrm{3,4} and5}$ denotes coefficients of the regressors and ${\varepsilon }_{t}$, ${\rho }_{t}$ and ${\sigma }_{t}$ denotes the error term in each model. For EKC hypothesis to hold in India, ${\beta }_{1}$(${\gamma }_{1}$) and ${\beta }_{2}$(${\gamma }_{2}$) must be positive and negative. Since LF is a measure of ecological quality, the LCC hypothesis will hold if ${\vartheta }_{1}$ is negative and ${\vartheta }_{2}$ is positive and statistically significant. Unlike the EKC hypothesis that acknowledged the inverted U-shaped association between environmental quality and income, the LCC hypothesis affirms the U-shaped association between ecological quality and income. Despite some research suggesting that nuclear energy intensify ecological deterioration^26,40^ some scholars^17,48^ have identified that nuclear energy intensity ecological quality. Since India is a global leader in the generation and consumption of nuclear energy, ${\beta }_{3}$(${\gamma }_{3}$) is anticipated to be negative while ${\vartheta }_{3}$ is expected to be positive. Moreover, human capital (HC) is critical to increasing EQ through increasing energy efficiency^30,44^. Furthermore, HC increases human productivity by improving the manufacturing process and increases economies' willingness to embrace energy-efficient and pollution-free technologies in the transportation, household, and industrial sectors. Therefore, ${\beta }_{4}$(${\gamma }_{4}$) is anticipated to be negative while ${\vartheta }_{4}$ is expected to be positive. Gas consumption in fossil fuel-based energy as a result, is consumption is expected to intensify ecological deterioration^46^. Therefore, ${\beta }_{5}$(${\gamma }_{5}$) is anticipated to be positive while ${\vartheta }_{5}$ is expected to be negative.

### Methodology

The combined cointegration test and ARDL are used in the research to examine the cointegration interrelationship and the connections between environmental proxies (LF, EF and CO_2_) and the regressors (GAS, EG, EGSQ, NC and HC) in the long and short-term. The ARDL bounds test method developed by^49^ facilitates the short- and long-run elasticities estimation simultaneously, as well as the investigation of the cointegration interrelationship between series with various order of integration i.e., (I(0) or I(1) mix). Furthermore, the ARDL procedure generates consistent outcomes in samples with few observations. The bounds test is conducted by setting up the UECM in Eqs. (4–6), and the cointegration investigation is executed by implementing the Wald test to the coefficients in the long-run.

4 $$\Delta {InCO}_{2t}= {\beta }_{0}+\sum_{i=1}^{a}{\beta }_{1i}\Delta In{CO}_{2t-i}+\sum_{i=1}^{b}{\beta }_{2i}\Delta In{EG}_{t-i}+\sum_{i=1}^{c}{\beta }_{3i}\Delta In{EGSQ}_{t-i}+\sum_{i=1}^{d}{\beta }_{4i}\Delta In{NC}_{t-i}+\sum_{i=1}^{e}{\beta }_{5i}\Delta In{HC}_{t-i}+\sum_{i=1}^{f}{\beta }_{6i}\Delta In{GAS}_{t-i}+ {\delta }_{1}{CO}_{2t-1}+ {\delta }_{2}{EG}_{t-1}+{\delta }_{3}{EGSQ}_{t-1}+{\delta }_{4}{NC}_{t-1}+{\delta }_{5}{HC}_{t-1}+ {\delta }_{6}{GAS}_{t-1}+{\varepsilon }_{t}$$

5 $$\Delta {InEF}_{t}= {\gamma }_{0}+\sum_{i=1}^{a}{\gamma }_{1i}\Delta In{EF}_{t-i}+\sum_{i=1}^{b}{\gamma }_{2i}\Delta In{EG}_{t-i}+\sum_{i=1}^{c}{\gamma }_{3i}\Delta In{EGSQ}_{t-i}+\sum_{i=1}^{d}{\gamma }_{4i}\Delta In{NC}_{t-i}+\sum_{i=1}^{e}{\gamma }_{5i}\Delta In{HC}_{t-i}+\sum_{i=1}^{f}{\gamma }_{6i}\Delta In{GAS}_{t-i}+ {\tau }_{1}{EF}_{t-1}+ {\tau }_{2}{EG}_{t-1}+{\tau }_{3}{EGSQ}_{t-1}+{\tau }_{4}{NC}_{t-1}+{\tau }_{5}{HC}_{t-1}+ {\tau }_{6}{GAS}_{t-1}+{\rho }_{t}$$

6 $$\Delta {InLF}_{t}= {\vartheta }_{0}+\sum_{i=1}^{a}{\vartheta }_{1i}\Delta In{LF}_{t-i}+\sum_{i=1}^{b}{\vartheta }_{2i}\Delta In{EG}_{t-i}+\sum_{i=1}^{c}{\vartheta }_{3i}\Delta In{EGSQ}_{t-i}+\sum_{i=1}^{d}{\vartheta }_{4i}\Delta In{NC}_{t-i}+\sum_{i=1}^{e}{\vartheta }_{5i}\Delta In{HC}_{t-i}+\sum_{i=1}^{f}{\vartheta }_{6i}\Delta In{GAS}_{t-i}+ {\pi }_{1}{LF}_{t-1}+ {\pi }_{2}{EG}_{t-1}+{\pi }_{3}{EGSQ}_{t-1}+{\pi }_{4}{NC}_{t-1}+{\pi }_{5}{HC}_{t-1}+ {\pi }_{6}{GAS}_{t-1}+{\sigma }_{t}$$

In Eq. (4), ${\beta }_{0}$ denotes intercept, the coefficients of short-long-run are denoted by ${\beta }_{\mathrm{1,2},\mathrm{3,4} 5 and 6}$ and ${\delta }_{\mathrm{1,2},\mathrm{3,4}, 5 and 6}$. In Eq. (5), ${\gamma }_{0}$ denotes intercept, the short-long-run coefficients are denoted by ${\gamma }_{\mathrm{1,2},\mathrm{3,4} and 5}$ and ${\tau }_{\mathrm{1,2},\mathrm{3,4} 5 and 6}$. In Eq. (6), ${\vartheta }_{0}$ denotes intercept, the short-long-run coefficients are denoted by ${\vartheta }_{\mathrm{1,2},\mathrm{3,4} 5 and 6}$ and ${\vartheta }_{\mathrm{1,2},\mathrm{3,4} 5 and 6}$. Moreover, a, b, c, d, and e are the lag length while the error terms are shown by a, b, c, d, e and f respectively. For the existence of a cointegration test, constraints are implemented to the lags of the explanatory indicators and constant term centred on case II (restricted and trend and intercept). For the existence of a cointegration test, constraints are implemented to the lags of the explanatory indicators and constant term centred on case II (restricted no trend and intercept). By dismissing the ${H}_{0}={H}_{1}={H}_{2}={H}_{3}={H}_{4}={H}_{5}={H}_{6}=0$, we dismiss the Ho hypothesis; therefore, there is an indication of long-run association. The flow of the analysis is shown below in Fig. 3.

## Findings and discussion

### Prerequisite results

**Figure 4:**
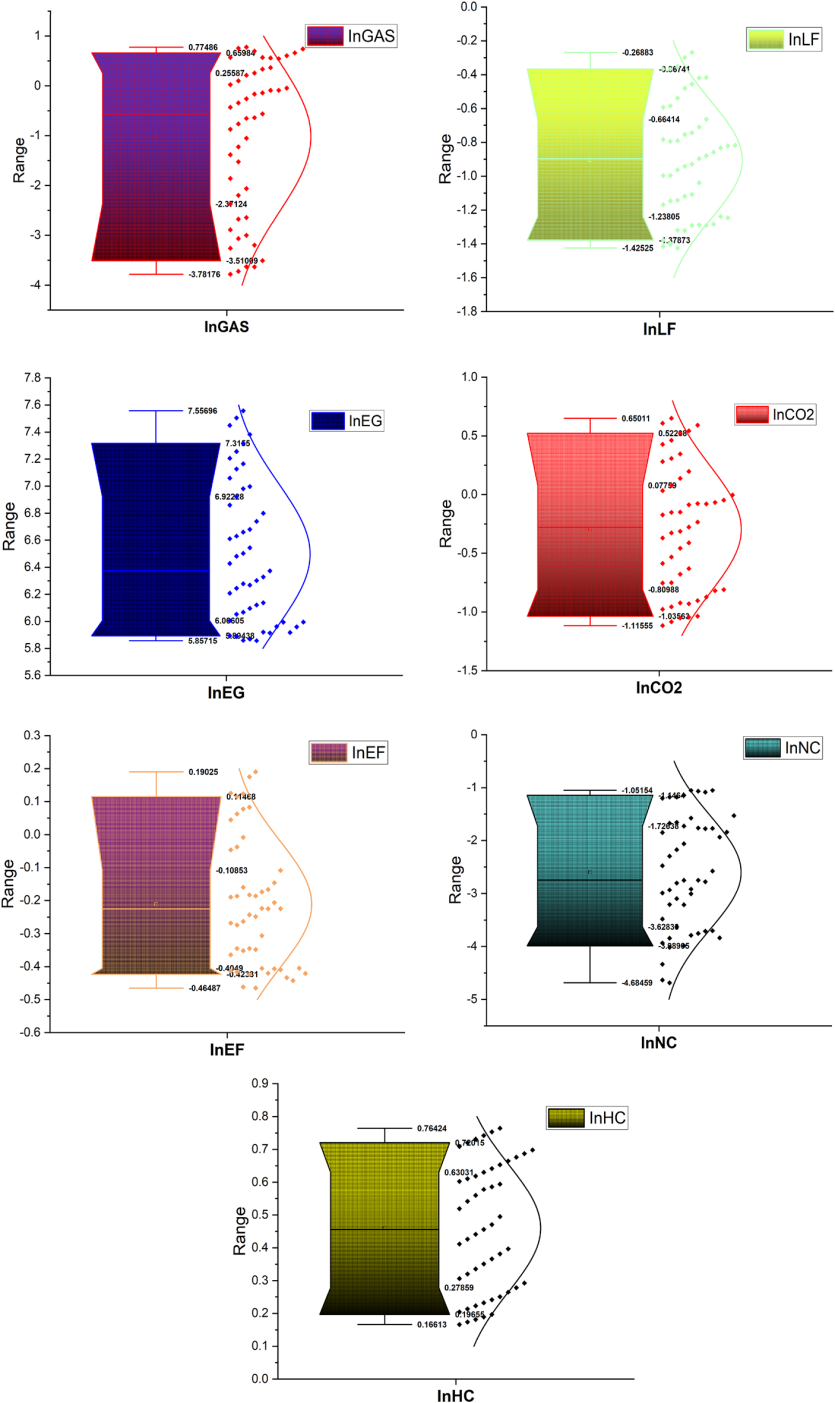
Box Plot of InEF, InEG, InHC, InLF, InCO_2_, InGAS and InNC.

Table 2 portrays the brief data information. The mean of InEF, InEG, InGAS, InHC, InLF, InCO_2_ and InNC are 0.2113, 6.4973, − 1.0205, 0.4601, − 0.9048, − 0.2958 and − 2.6004. The standard deviation values disclose that InEF is less volatile while InNC is highly volatile. The skewness value uncovers that InEF, InEG, InLF, InCO_2_ and InNC are positively skewed while InGAS and InHC are skewed positively. Besides, all the series conform with normal distribution as demonstrated by the kurtosis results. In addition, kurtosis results unearth that all the series are platykurtic. Furthermore, the study used the QQ plot to show the pictorial information of the series of study (Fig. 4).

**Table 2:** Descriptive statistics.

	InEF	InEG	InGAS	InHC	InLF	InCO_2_	InNC
Mean	− 0.2113	6.4973	− 1.0205	0.4601	− 0.9048	− 0.2958	− 2.6004
Median	− 0.2249	6.3732	− 0.5606	0.4559	− 0.8969	− 0.2765	− 2.7482
Maximum	0.1902	7.5569	0.7748	0.7642	− 0.2688	0.6501	− 1.0515
Minimum	− 0.4648	5.8571	− 3.7817	0.1661	− 1.4252	− 1.1155	− 4.6845
Std. Dev	0.1932	0.5241	1.5153	0.1937	0.3468	0.5384	1.0746
Skewness	0.5675	0.4905	− 0.5316	− 0.0182	0.1857	0.1164	− 0.1542
Kurtosis	2.1872	1.9692	1.8350	1.5841	1.8962	1.8235	1.8364
Jarque–Bera	3.9792	4.1346	3.8788	4.0952	2.7689	2.9364	2.9582
Probability	0.1367	0.1265	0.1789	0.1290	0.2504	0.2303	0.2278

The current analysis proceeds by evaluating the stationarity properties of the series. Knowledge of the stationarity properties of the parameter is vital to determine the type of cointegration that will suit the study variables. As a result, both PP and ADF tests are used to scrutinize the series order of integration. Table 3 shows the PP and ADF test outcomes and we observed that with the immunity of InNUC which is stationary at level, the other series (i.e., InGAS, InEG, InCO_2_, InHC, InEF and InLF) exhibit unit root at level but stationary at first difference.

**Table 3:** Unit root test results.

Variables	ADF		PP	
	Trend	Trend and intercept	Trend	Trend and intercept
InCO_2_	1.260	− 1.927	1.133	− 2.074
InEF	1.345	− 2.087	1.769	− 1.915
InLF	0.967	− 2.498	1.313	− 2.412
InEG	0.905	− 0.877	− 1.963	− 2.195
InGAS	− 2.253	− 0.625	− 2.072	− 0.625
InNC	− 3.883**	− 3.808**	− 3.820**	− 3.853**
InHC	− 1.183	− 2.214	− 1.087	2.185
$\Delta$InCO_2_	− 6.528*	− 6.745*	− 6.599*	− 6.786*
$\Delta$InEF	− 8.547*	− 9.416*	− 8.409*	− 9.378*
$\Delta$InLF	− 9.163*	− 9.467*	− 9.137*	− 9.467*
$\Delta$InEG	6.525*	− 6.538*	10.93*	− 11.45*
$\Delta$InGAS	− 7.378*	− 8.012*	− 7.358*	− 8.064*
$\Delta$InNC	–	–	–	–
$\Delta$InHC	− 4.732*	− 4.700*	− 4.197*	− 4.109**

*, **, and *** denotes significance at 1%, 5% and 10%. $\Delta$ denotes first difference.

### Cointegration results

Before examining the interrelation between the series, it is crucial to assess the cointegration between them. Thus, the present investigation evaluates the long-run connection between ecological quality proxies (InCO_2_, InEF and InLF) and the regressors. The study used a combined cointegration test with results shown in Table 4. Based on the outcomes, the Ho hypothesis of “no cointegration” is dismissed at a 1% in the InLF, InEF and InCO_2_ models.

**Table 4:** Combined cointegration outcomes.

	EG-JOH	EG-JOH-BO-BDM	CV-EG-J	CV-EG-JB-B
InCO_2_	27.081*	49.339*	1%: 15.84	1%: 30.77
InEF	24.725*	44.526*	5%: 10.57	5%: 20.14
InLF	32.930*	61.825*	10%: 8.301	10%: 15.93

The asterisks * denote a 1% significance level.

**Figure 5:**
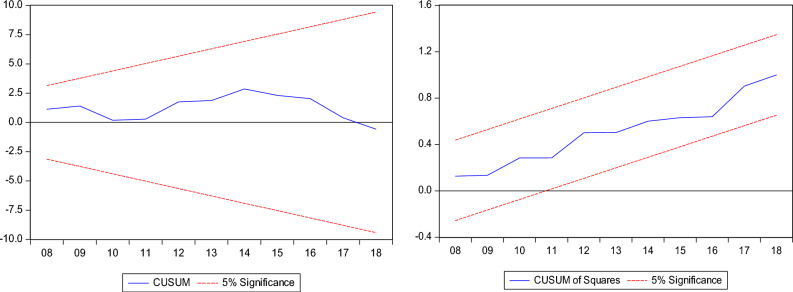
InCO_2_ model stability test result.

**Figure 6:**
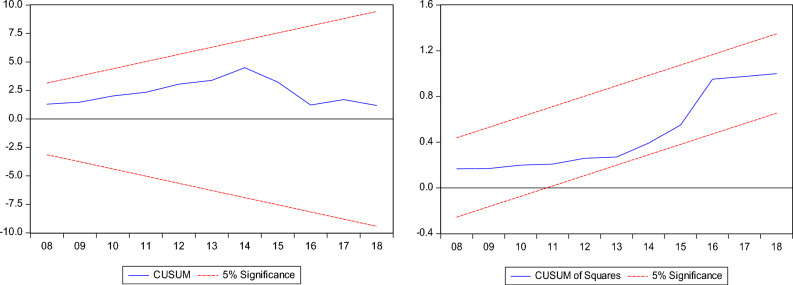
InCO_2_ model stability test result.

**Figure 7:**
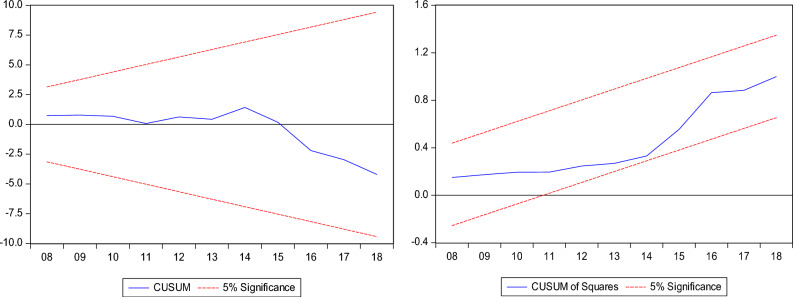
InCO_2_ model stability test result.

Furthermore, we employed the bounds test as a sturdiness assessment to the combined cointegration test with the results disclosed in Table 5. The bounds test result supports the dismissal of the null hypothesis of "no cointegration" in the three models which corroborates the combined cointegration results. Furthermore, we conduct post estimation tests, and the results show that the models have no issues with heteroscedasticity, autocorrelation, model specification and non-normal distribution. In addition, Figs. 5, 6 and 7 shows the stability test results of the InCO_2_, InEF and InLF models.

**Table 5:** Results for ARDL bounds test.

	InCO_2_	InEF	InLF
Dummy = global financial crisis	ARDL (1, 0, 2, 1, 0, 2)	ARDL (1, 1, 0, 1, 1, 0)	ARDL (1, 1, 0, 0, 0, 1)
F-statistics	5.790*	4.490**	4.808**
Narayan (2005) CV	1%	5%	10%
I(0)	3.967	2.893	2.427
I(1)	5.455	4.000	3.395
Diagnostic check			
Jarque–Bera	1.581 (0.453)	1.286 (0.525)	0.574 (0.750)
Ramsey-Reset	0.812 (0.422)	0.244 (0.808)	1.418 (0.255)
ARCH	0.355 (0.553)	0.169 (0.674)	0.625 (0.768)
BG-LM	2.420 (0.105)	1.919 (0.164)	1.797 (0.152)

The asterisks * and ** denote 1% and 5% significance levels.

### Results of ARDL long and short estimations

We advance in checking the short and long-run effect of the regressors i.e., EG, EGSQ, GAS, HC and NC on the ecological degradation proxies (LF, EF and LF) using the ARDL approach (see Table 6). In all three models, the value of ECT (− 1) falls between 0 and − 1 showing that the three models are significant. Dummy2008 represents the period of global financial crisis with impact India economy severely. As the financial crisis progressed into a packed global economic slump, India was incapable of escaping the second round of negative consequences. The worldwide catastrophe wedged India in three ways: trade flows, exchange rates and financial markets. As a result of this, India witnesses a decline in EF and CO_2_. The reduction in production during the crisis aids in improving ecological integrity, which is similar to the findings of^11^, who discovered that the 1997 Asia financial crisis lowered South Korea's ecological degradation.

**Table 6:** ARDL results.

	InCO_2_		InEF		InLF	
	Coefficient	Prob	Coefficient	Prob	Coefficient	Prob
Long-run						
InEG	1.009***	0.084	0.752**	0.025	− 0.803**	0.030
InEGSQ	− 0.050**	0.049	− 0.047***	0.053	0.071**	0.050
InGAS	0.050**	0.014	0.028**	0.031	0.064*	0.000
InNC	− 0.012***	0.096	− 0.008**	0.014	0.009***	0.088
InHC	− 1.006***	0.075	− 0.728**	0.013	0.942***	0.072
Dummy08	− 0.003**	0.022	− 0.051	0.864	0.015**	0.032
Constant	− 1.157	0.301	− 4.194	0.069	0.853	0.754
Short-run						
InEG	0.629**	0.027	0.393	0.441	1.803*	0.006
InEGSQ	− 0.259**	0.047	0.015*	0.000	0.091***	0.061
InGAS	1.0642	0.116	0.028***	0.059	− 0.064*	0.000
InNC	− 0.006***	0.075	− 0.035	0.533	0.030**	0.011
InHC	− 0.124*	0.001	− 0.087***	0.061	0.942	0.355
Dummy08	− 0.103	0.698	− 0.005	0.478	0.875	0.163
ECT (− 1)	− 0.775*	0.000	− 0.906*	0.000	− 0.810*	0.000
Constant	1.032	0.090	− 2.035	0.000	0.853	0.000

The asterisks *, ** and *** denote 1%, 5% and 10% significance level.

Conciseness, we only converse the long-run estimates. In the long-term, the EKC hypothesis is valid for the EF and CO_2_ models. These results show that EG impact CO_2_ and EF positively while EGSQ impact EF and CO_2_ negatively. Every economy requires energy to thrive. Nevertheless, the use of fossil fuels, such as gas, contributes to ecological damage. Countless nations depend on cheap and fossil fuel energy sources in their early stages of development. As a consequence, the linkage between EF is positive. Nevertheless, as nations become more prosperous, they gravitate toward ecological sustainability. Thus, there is a U-shaped nexus between ecological pollutants proxies (EF and CO_2_) and income. The observed results fulfil with the works of Refs.^47,50,51^ who reported a U-shape income-pollution interrelationship. Our findings also support the recently initiated load capacity curve (LCC) hypothesis, as the EG and EGSQ coefficients are both negative and positive respectively. Prior research has discovered a monotonically increasing connection between income and LF^52^. In contrast, this research suggests a U-shaped connection between LF and income.

The coefficient of nuclear energy (InNC) is significant and negative at a significance level of 10%. The result discloses that a 1% upsurge in InNC contributes to the mitigation of CO_2_ and EF by 0.012% and 0.008% respectively. Thus, InNC caused a reduction in ecological degradation. In the LF model, the coefficient of InNC is positive at a 10% level of significance suggesting that a 0.009% intensification in InLF is caused by 1% intensification of InNC. This finding indicates that augmented NC enhances India's quality of the environment. To put it another way, NC can be a reliable energy source that helps improve the general environment.

Likewise, the human capital (InHC) estimate is significantly negative. More specifically, a 1% intensification in InHC lessens CO_2_ by 1.006% and InEF by 0.728%, demonstrating that human capital aids in curbing ecological deterioration in India. In the InLF model, InHC improves and contributes to upsurges in InLF, indicating that a 1% upsurge in InHC boosts InLF by 0.942%. These findings show that HC can be credited to educated and skilled labour who are conscious of the dangers of a dirty ecosystem and practice eco-friendly methods. Environmental awareness and knowledge can favourably motivate ecological integrity by inspiring a sustainable and pro-environmental way of life. Furthermore, a knowledgeable and well-informed person prefers eco-friendly energy, which is necessary for a green ecosystem, energy conservation, innovation and energy security^30,35^.

Moreover, the gas consumption (InGAS) estimate is significantly positive in the InCO_2_ and InEF models. Therefore, a 1% intensification in InGAS intensifies CO_2_ by 0.050% and InEF by 0.028%, demonstrating that gas consumption intensifies ecological deterioration in India. In the InLF model, InGAS lessen InLF, indicating that a 1% upsurge in InGAS decreases InLF by 0.064%. These results are unsurprising given the fact that CO_2_, NOx and CH4 are emitted when natural gas and petroleum products are burned for cooking and heating (N_2_O). As per literature, gas usage is one of the leading causes of carbon emissions, which cause ecological deterioration and climate change^45,46^. Consequently, these findings are not surprising considering that carbon footprints account for a sizable portion of total ecological footprints.

### Robustness check

The current investigation employed the FMOLS (see Table 7) to evaluate the authenticity of the ARDL model results. The findings of the InCO_2_ and InEF models validate the EKC hypothesis. Furthermore, the InLF model validates the LCC hypothesis. Moreover, InGAS contribute to a decrease in ecological quality in the three models. Furthermore, InNC boosts the ecological quality of India as reported by the three models. Besides, InHC boosts ecological quality as shown in the three models. Lastly, the Dummy variables contribute to a decrease in ecological quality. All these results confirm the results obtained from ARDL long-run estimates.

**Table 7:** FMOLS results.

	InCO_2_		InEF		InLF	
	Coefficient	Prob	Coefficient	Prob	Coefficient	Prob
InEG	0.943**	0.020	0.650**	0.012	− 0.746***	0.071
InEGSQ	− 0.043**	0.011	− 0.037***	0.088	0.056***	0.091
InGAS	0.059*	0.000	0.040*	0.000	− 0.059*	0.000
InNC	− 0.018**	0.045	− 0.021**	0.014	0.011*	0.075
InHC	− 0.191**	0.038	− 0.554***	0.052	0.888***	0.042
Dummy08	− 0.002	0.859	0.011	0.299	0.014	0.272
Constant	0.928	0.597	− 2.240	0.171	0.059	0.976

*, **, and *** denotes significance at 1%, 5% and 10%.

### Causality results

**Figure 8:**
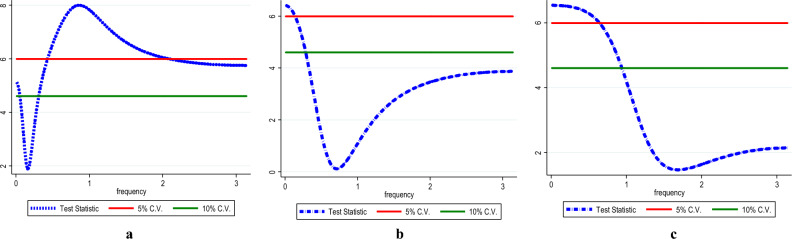
(**a**,**b**) InEG ≠ InCO2, (**c**) InEG ≠ InLF.

**Figure 9:**
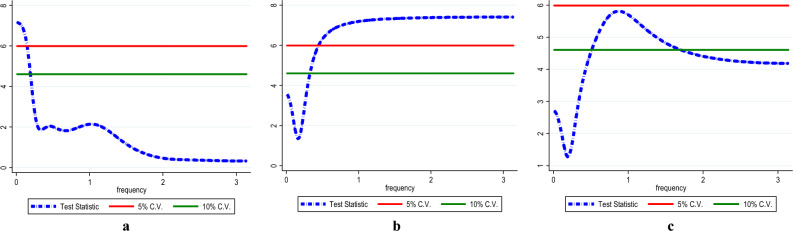
(**a**) InHC ≠ InCO2, (**b**) InHC ≠ InEF, (**c**) InHC ≠ InLF.

**Figure 10:**
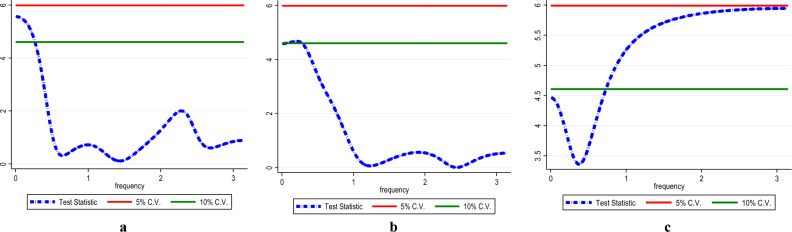
(**a**) InNC ≠ InCO2, (**b**) InNC ≠ InEF, (**c**) InNC ≠ InLF.

**Figure 11:**
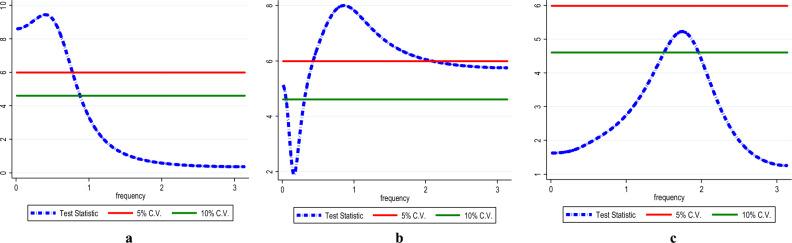
(**a**) InGAS ≠ InCO_2_, (**b**) InGAS ≠ InEF, (**c**) InGAS ≠ InLF.

The present study employed the frequency domain causality to identify the causality in all frequencies. Figure 8 shows the causality from InEG to InCO_2_ (Fig. 8a), InEF (Fig. 8b) and InLF (Fig. 8c) with the results affirming causality from InEG to InCO_2,_ InEF and InLF in the long-term. Similarly, there is causality running from InHC to InCO_2_ (see Fig. 9a), InEF (see Fig. 9b) and InLF (see Fig. 9c) in the long-term. Likewise, in the long-term causality is evident in the long-term from InNC to InCO_2_ (see Fig. 10a), InEF (see Fig. 10b) and InLF (see Fig. 10c). Lastly, we observed causality in the long-term from InGAS to InCO_2_ (see Fig. 11a), InEF (see Fig. 11b) and InLF (see Fig. 11c). The results infer that any policies regarding the variables at hand will significantly impact environmental quality in India.

### Discussion of findings

Global warming, energy justice and Climate change have emerged as major issues infuriating the international community (REF). Our study affirms the EKC and LCC hypothesis suggesting that at the initial phase economic growth will lessen the ecological quality; however, after a threshold is reached, economic growth will boost ecological quality. The studies of Refs.^11,52,53^ affirm the EKC and LCC hypotheses respectively.

Our results reveal that nuclear energy can play a significant part in decreasing the EF and CO_2_ as well as improving LF, implying that it is an eco-friendly energy source. This notion is supported by the majority of previous studies^23,48^. Energy, along with labour and capital, is a critical input in the manufacturing process^36^. Alternative sources of energy, especially NC, could be critical in holding the globe green and clean in such a situation^20^. Currently, nuclear energy accounts for 10% of total electricity production. Nuclear energy is critical for meeting energy demands while also enhancing ecological sustainability. According to figures, the use of universal nuclear energy has lowered energy-related pollution emissions by 10%^40^. For instance, in OECD nations, nuclear energy has reduced significantly the ecological damage induced by the electricity sector.

Furthermore, numerous investigations^19,20^ have discovered that the increasing role of nuclear energy is tightly connected to ecological quality. On the one hand, enhanced nuclear energy production ensures energy security by reducing reliance on foreign oil, the price of which is extremely volatile^54^. On the flip side, nuclear energy usage boosts economic growth while also improving ecological excellence. Although nuclear energy is crucial for improving ecological integrity, its effectiveness will be determined by the country's economic categorization and socio-economic factors that complement the energy policy to achieve sustainable development^15^. On the one hand, nuclear energy has large market prospects; additionally, this type of energy is cost-efficient. Besides enhancing ecological integrity, advancement in nuclear energy can ensure the security of energy and stimulate economic growth^23^. Besides, nuclear energy-inspired technological innovations can be critical in resolving economic, social, and environmental issues^40^.

Another substantial finding of the study is that human capital plays a beneficial role in enhancing ecological quality. Human capital has a beneficial role on the ecosystem because it is the result of training, education, research and experience, all of which are essential production function inputs. Scholars have argued that there is a positive interrelationship between per capita income and education which increases renewable energy demand and ecological sustainability^55^. We can deduce from a close examination of the literature on the interrelationship between economic growth and human capital that most countries have embraced skilled-oriented production strategies that have assisted them in achieving long-term growth^41^. Furthermore, increasing societal education can raise ecological consciousness by boosting superior ecological regulations such as recycling, energy-efficient appliances, water and energy conservation and the execution of emissions mitigation measures^56^. All of these factors contribute to the reduction of ecological damage in India. This outcome also suggests that Indian policymakers can invest in both human capital and ecological sustainability at the same time.

In terms of gas usage and economic growth, both are harmful to the environment. Energy is vital to a nation's economic progress; nevertheless, gas consumption continues to contribute considerably to the globe's energy source generation. Consumption of natural gas accounts for 79% of direct fossil fuel CO_2_ emissions from the commercial and residential sectors in 2020. Besides, India is ranked 14th in the worldwide in terms of natural gas consumption, consuming approximately 1.5% of total global consumption of 132,290,211 MMcf.

Moreover, an increasing body of evidence agrees that nonrenewable energy sources is a significant contributor to ecological deterioration^45,57,58^. Furthermore, existing studies concede that energy is critical to a nation's economic progress^7,59^. Nevertheless, most countries, including India, remain reliant strongly on fossil fuel sources of energy for their energy demands, which is a critical factor in stimulating economic progress and consequently, contributes to ecological degradation^14,53^.

## Conclusion and policy recommendations

### Conclusion

An upsurge in anthropogenic operations has caused mutilation to the ecosystem, causing land erosion, climate change, biodiversity loss and contamination. There is a wealth of research that has examined the various drivers of ecological quality in various countries and regions. Human capital and nuclear energy are both seen to be eco-friendly. Nevertheless, most previous studies have concentrated on carbon emissions and ecological footprint as ecological deterioration proxies, which no longer represent the whole environment. The EF only captures the demand side of the ecosystem. Therefore, in line with the studies of Refs.^21,53^ we used the load capacity factor (LF) which integrates both the demand and supply side of the environment. Given the advantage of LF over EF and CO_2_, we used LF as an ecological quality gauge. In addition, both EF and CO_2_ are also considered in this study as a proxy for ecological damage. Therefore, the study examines the effect of nuclear energy and gas consumption on ecological quality/degradation proxies (LF, EF and CO_2_) in India using data between 1970 and 2018. The results from this study affirm the LLC and EKC hypotheses for the case of India. Furthermore, the 2008 global financial crisis has had a positive effect on ecological quality. Besides, economic growth and gas consumption decrease ecological quality while nuclear energy and human capital boost ecological quality. Furthermore, the frequency domain causality discloses that all the regressors can predict ecological quality/degradation in India mostly in the long term.

### Policy recommendation

The research’s results are important because they affirm the long-run interrelationship between the variables of concern. Based on research outcomes, we can now make some significant policy recommendations to the stakeholders involved. According to the findings, nuclear energy can help trigger EQ in India. As a result, India has used nuclear energy to constrain ecological damage to a tolerable level. Based on the IEA (2020), nuclear energy accounts for 10.1% of global electricity production. India is ranked 14th globally in terms of nuclear-generating countries. Furthermore, nuclear energy is a dependable and inexhaustible energy source that increases global energy security. Moreover, because it is carbon-free, it may considerably add to a green and clean ecosystem. As a result, boosting nuclear energy production ought to be part of all nations' environmental and energy plans to achieve global energy security and a healthier climate.

Furthermore, it is obvious from India's footprint that the country is heavily reliant on fossil fuel energy, and as a consequence, it should boost the proportion of renewable and sustainable energy sources, notably nuclear energy, in its entire energy mix. This will enable the achievement of SDGs 7 and 13. Moreover, human capital may significantly contribute to environmental preservation; thus, Indian businesses and organizations should embrace the human-capital-intensive process of production. Human capital-led production practices will use less energy and physical resources, thus benefitting the ecosystem. Human capital, encompassing education, training, professionalism, and experience, plays a pivotal role in addressing ecological challenges. Therefore, policymakers should prioritize initiatives aimed at expanding literacy rates and improving professional skills as integral components of ecological protection efforts. In addition to formal education, fostering environmental awareness and consciousness should be promoted through collaborations with civil society organizations. To effectively mitigate the consequences of climate change, decision-makers must adopt comprehensive measures that integrate multiple environmental and social dimensions. This includes incorporating cultural norms, recognizing the intrinsic value of life and nature, fostering consumer responsibility, and promoting ecological literacy among the population.

In future research, it is crucial that the nuclear energy-environment nexus is explored in a broader range of developing and developed countries. While our study primarily focused on linear analysis, it is important to note that non-linear research has gained importance in recent years, as many macroeconomic phenomena exhibit asymmetric patterns. Therefore, considering non-linear dynamics should be a priority in future investigations to enhance our understanding of the complexities associated with the nuclear energy-environment relationship.

## Data Availability

All data generated or analysed during this study are available on reasonable request from the corresponding author.

## Abbreviations

ARDL: Autoregressive distributed lag
CO_2_: Carbon emissions
EF: Ecological footprint
EKC: Environmental Kuznets curve
GDP: Economic growth
HC: Human capital
LF: Load capacity factor
LCC: Load capacity curve
NGAS: Natural gas consumption
SDGs: Sustainable development goals

## References

[1] Das S, Nayak J, Naik B (2023). An impact study on COVID-19 and tourism sustainability: Intelligent solutions, issues and future challenges. World Rev. Sci. Technol. Sustain. Dev..

[2] Ali HS (2023). Structural transformations and conventional energy-based power utilization on carbon emissions: Empirical evidence from Pakistan. Environ. Dev. Sustain..

[3] Gupta, M., Saini, S. & Sahoo, M. Determinants of ecological footprint and PM2.5: Role of urbanization, natural resources and technological innovation. *Environ. Challenges* **7**, 100467 (2022).

[4] Alola, A. A. & Adebayo, T. S. Are green resource productivity and environmental technologies the face of environmental sustainability in the Nordic region? *Sustain. Dev.* **2**, (2022).

[5] Bekun FV, Emir F, Sarkodie SA (2019). Another look at the relationship between energy consumption, carbon dioxide emissions, and economic growth in South Africa. Sci. Total Environ..

[6] Lv Z (2017). The effect of democracy on CO2 emissions in emerging countries: Does the level of income matter?. Renew. Sustain. Energy Rev..

[7] Ozturk I, Acaravci A (2016). Energy consumption, CO2 emissions, economic growth, and foreign trade relationship in Cyprus and Malta. Energy Sour. Part B.

[8] Qi T, Zhang X, Karplus VJ (2014). The energy and CO2 emissions impact of renewable energy development in China. Energy Policy.

[9] Grossman GM, Krueger AB (1995). Economic growth and the environment*. Q. J. Econ..

[10] Siche R, Pereira L, Agostinho F, Ortega E (2010). Convergence of ecological footprint and emergy analysis as a sustainability indicator of countries: Peru as case study. Commun. Nonlinear Sci. Numer. Simul..

[11] Pata UK, Kartal MT (2022). Impact of nuclear and renewable energy sources on environment quality: Testing the EKC and LCC hypotheses for South Korea. Nucl. Eng. Technol..

[12] Sahoo M, Sahoo J (2022). Effects of renewable and non-renewable energy consumption on CO2 emissions in India: Empirical evidence from disaggregated data analysis. J. Public Aff..

[13] Sahoo M, Gupta M, Srivastava P (2021). Does information and communication technology and financial development lead to environmental sustainability in India? An empirical insight. Telemat. Inf..

[14] Shahbaz M, Hye QMA, Tiwari AK, Leitão NC (2013). Economic growth, energy consumption, financial development, international trade and CO2 emissions in Indonesia. Renew. Sustain. Energy Rev..

[15] Lin B, Moubarak M (2014). Renewable energy consumption—Economic growth nexus for China. Renew. Sustain. Energy Rev..

[16] Knight KW, Schor JB (2014). Economic growth and climate change: A cross-national analysis of territorial and consumption-based carbon emissions in high-income countries. Sustainability.

[17] Evidence from panel Granger causality tests (2016). Saidi, K. & Ben Mbarek, M. Nuclear energy, renewable energy, CO2 emissions, and economic growth for nine developed countries. Prog. Nucl. Energy.

[18] Rout SK, Gupta M, Sahoo M (2022). The role of technological innovation and diffusion, energy consumption and financial development in affecting ecological footprint in BRICS: An empirical analysis. Environ. Sci. Pollut. Res..

[19] Irfan, M., Sunday Adebayo, T., Cai, J., Dördüncü, H. & Shahzad, F. Analyzing the mechanism between nuclear energy consumption and carbon emissions: Fresh insights from novel bootstrap rolling-window approach. *Energy Environ.* **4**, 0958305X221133260 (2022).

[20] Menyah K, Wolde-Rufael Y (2010). CO2 emissions, nuclear energy, renewable energy and economic growth in the US. Energy Policy.

[21] Pata UK, Samour A (2022). Do renewable and nuclear energy enhance environmental quality in France? A new EKC approach with the load capacity factor. Prog. Nucl. Energy.

[22] Saidi K, Omri A (2020). Reducing CO2 emissions in OECD countries: Do renewable and nuclear energy matter?. Prog. Nucl. Energy.

[23] Wang, C., Raza, S. A., Adebayo, T. S., Yi, S. & Shah, M. I. The roles of hydro, nuclear and biomass energy towards carbon neutrality target in China: A policy-based analysis. *Energy* 125303. 10.1016/j.energy.2022.125303 (2022).

[24] Das N (2023). Can clean energy adoption and international trade contribute to the achievement of India’s 2070 carbon neutrality agenda? Evidence using quantile ARDL measures. Int. J. Sust. Dev. World.

[25] Pan, B., Adebayo, T. S., Ibrahim, R. L. & Al-Faryan, M. A. S. Does nuclear energy consumption mitigate carbon emissions in leading countries by nuclear power consumption? Evidence from quantile causality approach. *Energy Environ.* 0958305X221112910. 10.1177/0958305X221112910 (2022).

[26] Baek J, Pride D (2014). On the income—nuclear energy—CO2 emissions nexus revisited. Energy Econ..

[27] Gangopadhyay P (2023). Revisiting the carbon pollution-inhibiting policies in the USA using the quantile ARDL methodology: What roles can clean energy and globalization play?. Renew. Energy.

[28] Jabari MS, Aga M, Samour A (2022). Financial sector development, external debt, and Turkey’s renewable energy consumption. PLoS ONE.

[29] Ahmed Z, Asghar MM, Malik MN, Nawaz K (2020). Moving towards a sustainable environment: The dynamic linkage between natural resources, human capital, urbanization, economic growth, and ecological footprint in China. Resour. Policy.

[30] Danish Hassan, S. T., Baloch, M. A., Mahmood, N. & Zhang, J. Linking economic growth and ecological footprint through human capital and biocapacity. *Sustain. Cities Soc.* **47**, 101516 (2019).

[31] Ahmad M (2023). Households’ perception-based factors influencing biogas adoption: Innovation diffusion framework. Energy.

[32] The impact of inflation dynamics (2022). Bilal, Khan, I., Tan, D., Azam, W. & Tauseef Hassan, S. Alternate energy sources and environmental quality. Gondwana Res..

[33] Khan I, Hou F (2021). The impact of socio-economic and environmental sustainability on CO2 emissions: A novel framework for thirty IEA countries. Soc. Indic. Res..

[34] Huang S-Z, Chien F, Sadiq M (2022). A gateway towards a sustainable environment in emerging countries: The nexus between green energy and human Capital. Econ. Res. Ekonomska Istraživanja.

[35] Li X, Ullah S (2022). Caring for the environment: How CO2 emissions respond to human capital in BRICS economies?. Environ. Sci. Pollut. Res..

[36] Jayanthakumaran K, Verma R, Liu Y (2012). CO2 emissions, energy consumption, trade and income: A comparative analysis of China and India. Energy Policy.

[37] Kirikkaleli D, Adebayo TS (2021). Do public-private partnerships in energy and renewable energy consumption matter for consumption-based carbon dioxide emissions in India?. Environ. Sci. Pollut. Res..

[38] Udemba EN, Güngör H, Bekun FV, Kirikkaleli D (2021). Economic performance of India amidst high CO2 emissions. Sustain. Prod. Consum..

[39] Murshed M, Saboori B, Madaleno M, Wang H, Doğan B (2022). Exploring the nexuses between nuclear energy, renewable energy, and carbon dioxide emissions: The role of economic complexity in the G7 countries. Renew. Energy.

[40] Usman A, Ozturk I, Naqvi SMMA, Ullah S, Javed MI (2022). Revealing the nexus between nuclear energy and ecological footprint in STIRPAT model of advanced economies: Fresh evidence from novel CS-ARDL model. Prog. Nucl. Energy.

[41] Yao Y, Ivanovski K, Inekwe J, Smyth R (2020). Human capital and CO2 emissions in the long run. Energy Econ..

[42] Mahmood N, Wang Z, Hassan ST (2019). Renewable energy, economic growth, human capital, and CO2 emission: An empirical analysis. Environ. Sci. Pollut. Res..

[43] Rahman MM, Nepal R, Alam K (2021). Impacts of human capital, exports, economic growth and energy consumption on CO2 emissions of a cross-sectionally dependent panel: Evidence from the newly industrialized countries (NICs). Environ. Sci. Policy.

[44] Ahmed Z, Nathaniel SP, Shahbaz M (2021). The criticality of information and communication technology and human capital in environmental sustainability: Evidence from Latin American and Caribbean countries. J. Clean. Prod..

[45] Dong K, Sun R, Li H, Liao H (2018). Does natural gas consumption mitigate CO2 emissions: Testing the environmental Kuznets curve hypothesis for 14 Asia-Pacific countries. Renew. Sustain. Energy Rev..

[46] Çıtak F, Uslu H, Batmaz O, Hoş S (2021). Do renewable energy and natural gas consumption mitigate CO2 emissions in the USA? New insights from NARDL approach. Environ. Sci. Pollut. Res..

[47] Pata UK (2018). Renewable energy consumption, urbanization, financial development, income and CO2 emissions in Turkey: Testing EKC hypothesis with structural breaks. J. Clean. Prod..

[48] Pan, B., Adebayo, T. S., Ibrahim, R. L. & Al-Faryan, M. A. S. Does nuclear energy consumption mitigate carbon emissions in leading countries by nuclear power consumption? Evidence from quantile causality approach. *Energy Environ.* 0958305X221112910. 10.1177/0958305X221112910 (2022).

[49] Pesaran MH, Shin Y, Smith RJ (2001). Bounds testing approaches to the analysis of level relationships. J. Appl. Economet..

[50] Al-Mulali U, Solarin SA, Ozturk I (2016). Investigating the presence of the environmental Kuznets curve (EKC) hypothesis in Kenya: An autoregressive distributed lag (ARDL) approach. Nat. Hazards.

[51] Jahanger A (2022). Impact of globalization on CO2 emissions based on EKC hypothesis in developing world: The moderating role of human capital. Environ. Sci. Pollut. Res..

[52] Yilanci V, Pata UK (2020). Investigating the EKC hypothesis for China: The role of economic complexity on ecological footprint. Environ. Sci. Pollut. Res..

[53] Adebayo, T. S. Environmental consequences of fossil fuel in Spain amidst renewable energy consumption: A new insights from the wavelet-based Granger causality approach. *Int. J. Sustain. Dev. World Ecol.* **1**, 1–14 (2022).

[54] Abbasi F, Riaz K (2016). CO2 emissions and financial development in an emerging economy: An augmented VAR approach. Energy Policy.

[55] Barro RJ (1991). Economic growth in a cross section of countries. Q. J. Econ..

[56] Holdren JP, Ehrlich PR (1974). Human Population and the Global Environment: Population growth, rising per capita material consumption, and disruptive technologies have made civilization a global ecological force. Am. Sci..

[57] Alola AA, Akadiri SS, Usman O (2021). Domestic material consumption and greenhouse gas emissions in the EU-28 countries: Implications for environmental sustainability targets. Sustain. Dev..

[58] Salazar-Núñez HF, Venegas-Martínez F, Lozano-Díez JA (2021). Assessing the interdependence among renewable and non-renewable energies, economic growth, and CO2 emissions in Mexico. Environ. Dev. Sustain..

[59] Acheampong AO, Dzator J, Shahbaz M (2021). Empowering the powerless: Does access to energy improve income inequality?. Energy Econ..

